# Expansion of HIV Preexposure Prophylaxis to 35 PEPFAR-Supported Early Program Adopters, October 2016–September 2018

**DOI:** 10.15585/mmwr.mm6908a3

**Published:** 2020-02-28

**Authors:** Gaston Djomand, Trista Bingham, Irene Benech, Mercy Muthui, Helen Savva, Stella Alamo, Chomnad Manopaiboon, Tisha Wheeler, Sasha Mital

**Affiliations:** ^1^Division of Global HIV and TB, Center for Global Health, CDC; ^2^U.S. Agency for International Development, Washington, D.C.

The U.S. President’s Emergency Plan for AIDS Relief (PEPFAR), the largest bilateral funder of human immunodeficiency virus (HIV) prevention and control programs worldwide, currently supports implementation of preexposure prophylaxis (PrEP) to reduce HIV incidence among persons at substantial risk for infection, including female sex workers, men who have sex with men (MSM), and transgender women (hereafter referred to as key populations). Recent estimates suggest that 54% of all global new HIV infections in 2018 occurred among key populations and their sexual partners (*1*). In 2016, PEPFAR began tracking initiation of PrEP by key populations and other groups at high risk (*2*). The implementation and scale-up of PrEP programs across 35 PEPFAR-supported country or regional programs* was assessed by determining the number of programs reporting any new PrEP clients during each quarter from October 2016 to September 2018. As of September 2018, only 15 (43%) PEPFAR-supported country or regional programs had implemented PrEP programs; however, client volume increased by 3,351% over the assessment period in 15 country or regional programs. Scale-up of PrEP among general population clients (5,255%) was nearly three times that of key population clients (1,880%). Among key populations, the largest increase (3,518%) occurred among MSM. Factors that helped drive the success of these PrEP early adopter programs included initiation of national, regional, and multilateral stakeholder meetings; engagement of ministries of health and community advocates; revision of HIV treatment guidelines to include PrEP; training for HIV service providers; and establishment of drug procurement policies. These best practices can help facilitate PrEP implementation, particularly among key populations, in other country or regional programs to reduce global incidence of HIV infection.

International, national, subnational, nongovernmental, and academic PEPFAR implementing partners reported PrEP data from country or regional programs for eight quarters (i.e., October–December 2016 through July–September 2018). The primary outcome measured was the number of persons (including new enrollees) who received PrEP during the reporting period, disaggregated by key population group (i.e., female sex workers, MSM, and transgender women). To assess implementation and scale-up of PrEP, the number and percentage of the 35 PEPFAR-funded country or regional programs that reported PrEP initiation among key populations or the general population and the number of new PrEP clients in each program were determined. The relative percentage change in PrEP initiation for the general population and that in the key populations over time were also compared.

Country or regional programs with >150 new key population clients across multiple quarters totaling ≥1,000 clients were classified as early adopters of PrEP among key populations. The threshold of 150 clients per quarter was determined by a minimum enrollment rate of 50 key population clients per month during a quarter. Using previous analyses of PrEP implementation (*3*,*4*), critical factors and scale-up accelerators that facilitate early coverage and rapid growth among PrEP programs were identified. This list of factors was provided to implementing partners in the early adopter programs, who were asked to determine whether these factors were relevant to the growth of their respective programs.

During the analysis period, 35 PEPFAR-supported country or regional programs were assessed. Substantial scale-up of PrEP initiation among 15 of these programs took place, commencing with 888 new clients during October–December 2016 and ending with 30,644 during July–September 2018 (Figure), representing a 3,351% overall increase among general population and key population clients over the assessment period. Scale-up of PrEP initiation among the general population (5,255% increase) was nearly three times that among the key populations (1,880%), and the increases in PrEP initiation within the key populations substantially varied among key population groups (Table 1). For example, the largest increase in enrollment of new clients within the key populations occurred among MSM (3,518%), whereas the increase among transgender women was substantially lower (573%). The percentage of new key population clients among all new PrEP clients varied over the analysis period (range = 29%–56%) and was 32% in the most recent quarter (July–September 2018). Overall, among 35 PEPFAR-supported country or regional programs, 15^†^ (34%) have reported providing PrEP services to general population clients, and 13^§^ have reported providing PrEP services to key population clients.

**FIGURE:**
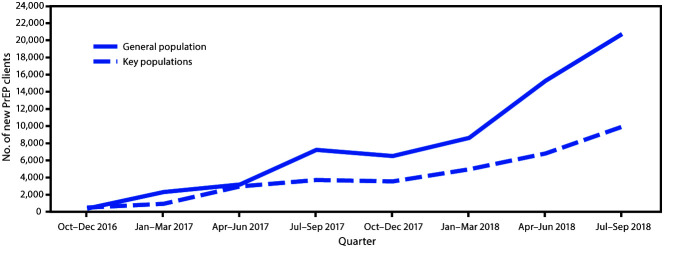
Preexposure prophylaxis (PrEP) initiation among the general population and key populations — 35 U.S. President’s Emergency Plan for AIDS Relief–funded country or regional programs, October 2016–September 2018 **Abbreviation:** AIDS = acquired immunodeficiency syndrome. * Female sex workers, men who have sex with men, and transgender women.

**TABLE 1:** Preexposure prophylaxis (PrEP) initiation among general and key populations and percentage change in PrEP initiation in general and key populations — 35 U.S. President’s Emergency Plan for AIDS Relief–funded country or regional programs, October 2016–September 2018

Characteristic	Quarter								% Increase Oct 2016–Sep 2018
	Oct–Dec 2016	Jan–Mar 2017	Apr–Jun 2017	Jul–Sep 2017	Oct–Dec 2017	Jan–Mar 2018	Apr–Jun 2018	Jul–Sep 2018	
**Total PrEP clients enrolled**	888	3,250	6,155	10,945	10,062	13,588	22,086	30,644	3,351
General population (%)*	387 (44)	2,308 (71)	3,188 (52)	7,228 (66)	6,509 (65)	8,621 (63)	15,277 (69)	20,723 (68)	5,255
Key populations (%)*	501 (56)	942 (29)	2,967 (48)	3,717 (34)	3,553 (35)	4,967 (37)	6,809 (31)	9,921 (32)	1,880
Female sex workers (%)^†^	390 (78)	709 (75)	1,470 (50)	2,125 (57)	2,075 (58)	3,106 (63)	4,098 (60)	6,553 (66)	1,580
Men who have sex with men (%)^†^	89 (18)	186 (20)	1,463 (49)	1,495 (40)	1,411 (40)	1,752 (35)	2,615 (38)	3,220 (32)	3,518
Transgender women (%)^†^	22 (4)	47 (5)	34 (1)	97 (3)	67 (19)	109 (2)	96 (1)	148 (1)	573
**No. of country or regional programs reporting PrEP initiation**	5	7	12	11	11	14	15	15	200
**No. of country or regional programs reporting PrEP initiation among key populations**	3	5	8	6	9	11	14	13	333

**Abbreviation:** AIDS = acquired immunodeficiency syndrome.

* Percentage of all PrEP clients.

^†^ Percentage of all key population clients.

Among all PEPFAR-supported programs, six (Asia Region, Kenya, South Africa, Uganda, Vietnam, and Zimbabwe) were classified as early adopters of PrEP for key populations. Implementing partners in five of these programs (all except Vietnam) identified critical factors for early adoption of PrEP (Table 2), including national and regional stakeholder meetings with strong ongoing engagement and advocacy from ministries of health, community advocates, and multilateral partners such as the World Health Organization and the Joint United Nations Programme on HIV/AIDS (UNAIDS). Five early adopter programs included PrEP services in national treatment and prevention guidelines despite the lack of favorable legal environments to safeguard key populations from violence and discrimination. Early programs also reported standardized, routine HIV service provider training and the ability of their governments to procure PrEP.

**TABLE 2:** Critical factors and scale-up accelerators associated with early adoption of expansion of preexposure prophylaxis (PrEP) among key populations in programs supported by the U.S. President’s Emergency Plan for AIDS Relief (PEPFAR) — Asia Region, Kenya, South Africa, Uganda, and Zimbabwe, October 2016-September 2018

Critical factors and scale-up accelerators	Asia Region	Kenya	South Africa	Uganda	Zimbabwe
**National stakeholder consultations and engagement**	National consultations on PrEP held in 2012	National and multilateral stakeholders meetings led by MOH in 2016	Extensive stakeholder consultations with academics and key population groups coordinated by NDoH in 2016	Multiple consultations including key population groups, MOH, and multilateral partners in 2017	Wide, multisectoral, multilateral stakeholder meetings including key population groups in 2016
**Favorable legal environment for key populations**	Not favorable but not criminalized	Not favorable; strong MOH advocacy for key populations and LGBT persons	Legal protection for LGBT persons	Not favorable	Not favorable
**Existing PrEP treatment guidelines**	National PrEP guidelines published in 2018	PrEP guidelines launched in July 2016	National policy on PrEP developed and published in March 2016	PrEP guidelines developed by MOH and endorsed in 2017	PrEP implementation plan and guidelines developed and launched in 2018
**HIV service provider training**	Training for health care workers at hospitals and health centers in 13 provinces with high HIV prevalence	Training, CME, and clinical mentorship for HIV service providers	Regular HIV service provider training for accredited PrEP centers	Training manual developed; training provided at 70 sites	Oral PrEP training curriculum developed and launched
**Existing drug procurement system**	National drug procurement system	National drug procurement system	PrEP procured by NDoH with CDC and PEPFAR funds	National drug procurement system	Funded through PEPFAR and pharmaceutical companies
**Provision of other services**	PrEP offered as part of general clinical services	PrEP integrated with behavioral and clinical services	Part of HIV combination prevention for all HIV service providers	Part of HIV combination prevention services for all HIV service providers	Part of HIV combination prevention services for all HIV service providers
**Government’s active ownership**	Development of web-based platform to monitor PrEP use	Prioritization of key populations for PrEP service delivery	NDoH coordination of PrEP rollout and data collection	Development of PrEP implementation tools and guidelines	National technical working group to oversee PrEP implementation
**Innovative demand creation activities**	Promoted through several social media channels, websites, and in key population safe spaces and meeting places	Promoted through national campaign, social media, and at key population clinics and meeting places	Done for each key population with social media and at drop-in centers and meeting places	Promoted through drop-in centers and the assistance of peers, peer educators, and peer navigators	Done for grassroots key population organizations in safe spaces and key population clinics
**Multiple service delivery models**	PrEP provided at eight key population–led service centers and in approximately 40 public hospitals and health centers	PrEP provided at drop-in centers, mobile outreach services	PrEP provided at drop-in centers and mobile clinics at key population meeting places	PrEP provided by outreach at drop-in centers and integrated into public facilities	PrEP provided at clinics and by mobile outreach

**Abbreviations:** CME = continuing medical education; HIV = human immunodeficiency virus; LGBT = lesbian, gay, bisexual, and transgender; MOH = ministry of health; NDoH = national department of health.

Factors that accelerated PrEP scale-up included active government ownership of the national PrEP program and innovations in PrEP service delivery. Most governments supported PrEP programs by drafting policies and guidelines, developing or adapting PrEP training curricula, accrediting sites, and collecting and reporting data. PrEP marketing innovations, including promoting PrEP outside the typical clinical platform through drop-in centers or key population safe spaces; on social media; at meeting places such as sex clubs and gay bars; and in the community through peer outreach also were critical accelerators (*5*).

## Discussion

PrEP implementation among key populations has been successful in some PEPFAR-supported programs despite the absence of laws and policies to protect key populations who seek HIV services. That key populations accounted for 29%–56% of all PrEP initiations underscores the relative success of PrEP implementation among these populations, given that they might represent a small proportion of the overall population. PrEP early adopter programs shared several critical characteristics and reported common scale-up accelerators, including cooperation among national governments, PrEP implementers, and community advocates. These factors can provide insights for programs that have not yet introduced or sufficiently scaled PrEP services to reach key populations.

As of October 2018, only 13 of 35 PEPFAR-supported country or regional programs were implementing PrEP for key populations. The findings in this report suggest that global and regional advocacy can help engage community stakeholders and encourage governments to develop national PrEP guidelines in programs that have yet to implement PrEP. For programs currently providing PrEP services, the scale-up accelerators identified in this report can help streamline services to key populations by integrating PrEP into a combination prevention strategy. For example, a successful PrEP program can attract persons seeking to learn their HIV status and subsequently identify HIV-negative persons at substantial risk for HIV, identify previously undiagnosed HIV-positive persons, and link patients with newly diagnosed HIV infection to rapid antiretroviral therapy initiation (*6*). This simultaneous delivery of PrEP and antiretroviral therapy services could synergize prevention, early identification of HIV-positive persons, and treatment to achieve epidemic control (*7*).

Because the effectiveness of PrEP depends upon consistent use among persons at continued risk, monitoring adherence to PrEP and retention in the program is important. Since 2019, PEPFAR has required programs to monitor PrEP adherence, retention, coverage, and potential impact (*2*). Novel PrEP delivery mechanisms (e.g., event-driven PrEP and long-acting PrEP injectable drugs) are being explored as alternatives to daily oral PrEP. One study in Thailand concluded that incorporating PrEP delivery into existing antiretroviral therapy programs could be a cost-effective strategy to prevent HIV infection among MSM (*5*).

The findings in this report are subject to at least two limitations. First, PrEP retention data were not collected; therefore, the impact of PrEP scale-up on epidemic control is unknown. Recognizing the need to quantify how long persons remain on PrEP, PEPFAR introduced a reporting indicator in October 2018 to track retention. Second, it is possible that, because of stigma, some participants did not disclose their status as members of key populations, leading to underestimation of the proportion of key populations initiating PrEP. To improve the correct classification of key population status among PrEP initiates, PEPFAR continues to support the efforts of country or regional programs to collect key population information that is equally important for clinical management of clients.

PrEP implementation in PEPFAR-supported country or regional programs is gradually increasing among general and key populations. Scale-up of this HIV prevention method in all populations at substantial risk and sharing best practices could contribute to faster HIV epidemic control. Cost-effectiveness and mathematical modeling studies might be useful to help identify subpopulations for PrEP delivery to achieve the greatest HIV prevention impact in resource-limited settings, including other PEPFAR programs.

**Summary**

**What is already known about this topic?**

Preexposure prophylaxis (PrEP) reduces human immunodeficiency virus (HIV) infection incidence.

**What is added by this report?**

During October 2016–September 2018, in 15 of 35 country or regional programs supported by the U.S. President’s Emergency Plan for AIDS Relief, the increase in PrEP coverage among the general population was approximately triple that among female sex workers, men who have sex with men, and transgender women. Critical factors associated with successful PrEP implementation in these populations include stakeholder engagement, existing PrEP delivery guidelines, HIV service provider training, and a drug procurement system.

**What are the implications for public health practice?**

Sharing best practices could facilitate adoption of PrEP in other country or regional programs and contribute to epidemic control.

## Footnotes

* Angola, Asia Region, Botswana, Burma, Burundi, Cambodia, Cameroon, Caribbean Region, Central America Region, Central Asia Region, Côte d'Ivoire, Democratic Republic of the Congo, Dominican Republic, Eswatini, Ethiopia, Ghana, Haiti, India, Indonesia, Kenya, Lesotho, Malawi, Mozambique, Namibia, Nigeria, Papua New Guinea, Rwanda, South Africa, South Sudan, Tanzania, Uganda, Ukraine, Vietnam, Zambia, and Zimbabwe.

† Asia Region, Botswana, Democratic Republic of the Congo, Dominican Republic, Kenya, Lesotho, Namibia, Nigeria, South Africa, Tanzania, Uganda, Ukraine, Vietnam, Zambia, and Zimbabwe. The Asia Region includes China, Laos, and Thailand.

§ Asia Region, Botswana, Dominican Republic, Kenya, Lesotho, Namibia, Nigeria, South Africa, Tanzania, Uganda, Vietnam, Zambia, and Zimbabwe.
